# Epithelial-Myoepithelial Carcinoma of the Base of Tongue with Possible Lung Metastases

**DOI:** 10.1155/2017/4973573

**Published:** 2017-09-20

**Authors:** Michael Y. Chen, Vipul Vyas, Ryan Sommerville

**Affiliations:** Royal Brisbane and Women's Hospital, Herston, QLD, Australia

## Abstract

**Background:**

Epithelial-myoepithelial carcinomas are rare neoplasms usually arising from the salivary glands. There is limited evidence in the literature on their prognosis in the base of the tongue but other cases have resolved without recurrence.

**Methods:**

The patient underwent biopsies demonstrating the diagnosis of epithelial-myoepithelial carcinoma of the base of tongue and a PET scan showed multiple bilateral rounded pulmonary nodules.

**Results:**

The patient declined chemotherapy and radiotherapy to maximise his quality of life and passed away under management from palliative care several months later.

**Discussion:**

This is the only case in the literature of this type of carcinoma in the base of the tongue resulting in metastases and a poor prognosis. The case highlights the importance of checking for metastases in such lesions and their potentially serious outcomes if left untreated.

## 1. Introduction

Epithelial-myoepithelial carcinoma is a rare neoplasm that usually arises from the salivary glands, comprising approximately 1% of all salivary gland tumours [1, 2]. Since it was first described in 1972, there have only been 566 cases of epithelial-myoepithelial carcinoma described in the literature. Histologically, the tumour is biphasic with clear myoepithelial cells surrounding epithelial-lined ducts resembling intercalated ducts [3]. The largest series of cases to date was a recent multi-institution review of 246 cases which showed a disease specific survival at 180 months of 80.7% [1]. The case presented is an uncommon presentation of epithelial-myoepithelial carcinoma of the base of the tongue with only four other cases found in the literature.

## 2. Case

Our case is of a 52-year-old gentleman who presented with restricted tongue movement, dysarthria, and dysphagia that developed progressively over several months. He mentioned this at the Ear Nose and Throat clinic during follow-up of surgical drainage of a mucocele from the pterygopalatine fossa six months before. He also reported 15 kg of weight loss in the preceding four months. He is a nonsmoker with no medical history except for osteomyelitis following a meniscal knee repair four years before.

**Figure 1:**
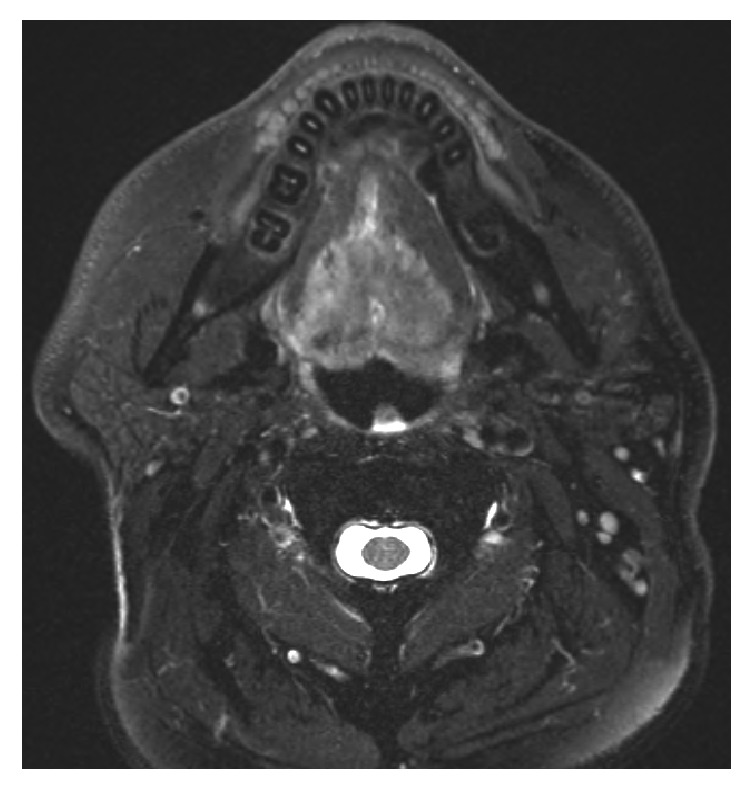
MRI of lesion in base of tongue.

An MRI showed a 37 × 34 × 42 mm mass in the base of tongue extending to the geniohyoid muscle as well as the genioglossus muscle that was most concerning for squamous cell carcinoma as shown in Figure 1.

His PET/CT scan showed a large intensely FDG avid tongue mass arising in the posterior of the tongue within the body of the tongue and extrinsic muscles and extending posterosuperiorly into the region of the lingual tonsils and midline tongue superiorly. There was moderate FDG uptake in adjacent nodes consistent with metastases.

Multiple bilateral rounded pulmonary nodules were present within the lungs bilaterally, the largest of which measured 5 mm. These showed no significant FDG uptake and were below the resolution of the PET/CT to accurately characterise.

The lung lesions were too small for biopsy to confirm metastasis; however, it was judged to be metastatic given the rapidly progressive disease and bilateral neck metastases. The tumour was staged as T4N2cM1.

**Figure 2:**
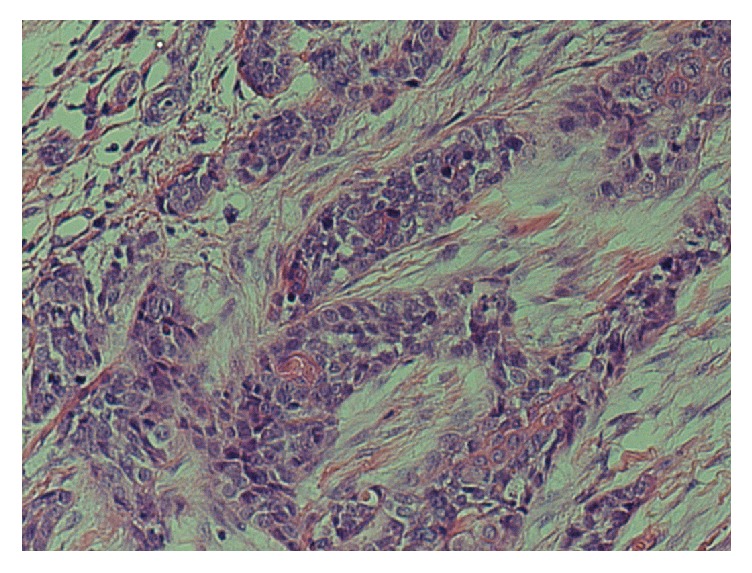
Haematoxylin and eosin (H&E) stain at ×40 magnification.

**Figure 3:**
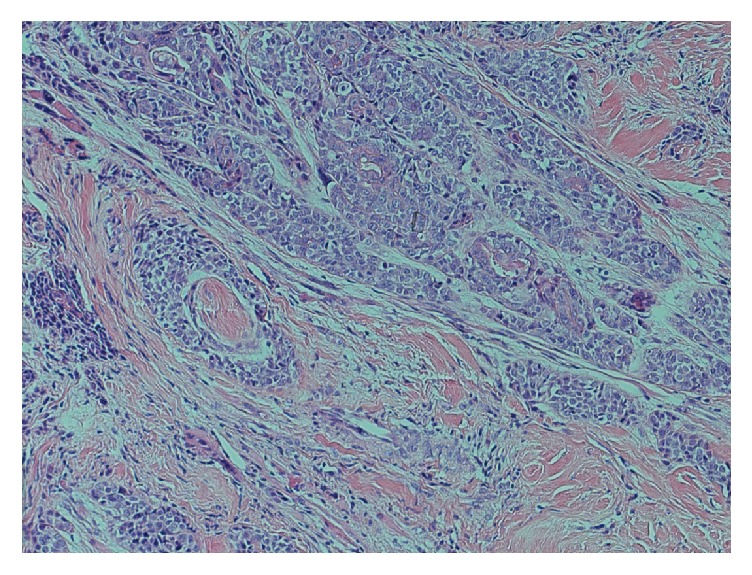
H&E stain demonstrating perineural invasion of the tumour.

Two tongue biopsies performed were inconclusive; however, the third biopsy a month later showed an infiltrating biphasic tumour with solid nests composed of epithelial and myoepithelial cells as shown in Figure 2. The tumour cells also demonstrated perineural invasion as shown in Figure 3. There was nuclear atypia and prominent mitoses present. Tumour cells were positive for SMA, p40, p63, CK5/6, EMA (patchy and luminal), CEA (luminal), and CD43. Ki-67 reveals a high proliferation index (60%). The diagnosis was epithelial-myoepithelial carcinoma with myoepithelial anaplasia.

Subsequently, the patient was offered radical local treatment with radiotherapy and chemotherapy. The patient declined these so as to maximise his quality of life. He was referred to the palliative care service for continued care and over a year later required a percutaneous endoscopic gastrostomy (PEG) tube for feeding. Approximately 18 months after the diagnosis, the patient passed away from obstruction of the airway.

## 3. Discussion

Epithelial-myoepithelial carcinoma is a rare low-grade neoplasm that was first characterised in 1972 [7]. They most commonly arise in the salivary glands with approximately 60–80% arising in the parotid glands [1, 3]. The mean age is around 60 and affects females at a ratio of 1.5 : 1 with a mean tumour size of approximately 29 mm [3].

Epithelial-myoepithelial carcinomas are primarily treated surgically, as shown in the largest study with all but 9 of the 207 patients undergoing surgery [1]. 85 of these patients (41.1%) received radiotherapy in addition to surgery; however, no survival benefit was noted for those who received radiotherapy compared to those who did not.

Epithelial-myoepithelial carcinomas do not commonly have distant metastases and this case presents the possibility of lung metastasis, although unfortunately histological confirmation was not obtained. In a review of 58 patients with epithelial-myoepithelial carcinomas, only 3 patients (5.2%) had evidence of metastatic disease with only one case being a distant metastasis to the iliac bone [3]. In the largest review of 246 cases of epithelial-myoepithelial carcinoma, 11 patients (4.47%) had distant metastases; however, the locations were unspecified. There is only one case in the literature of pulmonary metastases which were discovered 14 years after a parotidectomy for epithelial-myoepithelial carcinoma of the salivary gland [8].

The pathology in our case also showed a high proliferation index of 60% with Ki-67 staining. In contrast, a review of 61 epithelial-myoepithelial carcinomas found a mean proliferative index of 16.9% with a range from 0 to 50% [3]. There have been cases of epithelial-myoepithelial carcinoma dedifferentiating into a high-grade carcinoma (HGC) with a proliferation index of 67.1% in the HGC and 11.5% in the epithelial-myoepithelial portion [9].

It is also rare for epithelial-myoepithelial carcinoma to develop in the base of tongue with only 4 other cases in the literature to our knowledge [2, 4–6]. Two of these patients received surgery and two received chemoradiotherapy. Therefore, this is, to our knowledge, the first case of epithelial-myoepithelial carcinoma in the base of tongue that has not been aggressively treated and instead given palliative support.

A comparison between the known cases in the literature of epithelial-myoepithelial carcinoma in the tongue is summarised in Table 1 and is a continuation of the review done by Peters et al. [2].

Our case shows the potential malignancy of epithelial-myoepithelial carcinomas with distant metastasis to the lungs and lymph nodes. It is the second case in the literature of epithelial-myoepithelial carcinoma with metastases to the lungs and only the fifth case to occur in the tongue, to our knowledge. Although this is a rare presentation of the tumour, this case may help to guide patient decision-making and prognostic information in the future by demonstrating the potential severity of the disease.

## Figures and Tables

**Table 1:** Cases found in the literature of epithelial-myoepithelial carcinoma in the tongue.

	Puri et al. 2004 [4]	Kumai et al. 2006 [5]	Peters et al. 2010 [2]	de Matos et al. 2010 [6]	Chen et al. (2016)
Demographics	48 yo male	76 yo male	60 yo female	48 yo female	52 yo male

Tumour size	50 × 30 mm	40 × 20 mm	37 × 15 mm	10 mm	37 × 34 mm

Treatment	3-drug chemotherapy (cisplatin, doxorubicin, and 5-fluorouracil) Radiotherapy (66 Gy)	Surgery (subtotal glossectomy, bilateral neck dissection and rectus abdominis flap)	Radiotherapy, 60 Gy over 30 fractions, 2 cm margin	Excisional biopsy, but reoperated four years later due to recurrence	Refused chemotherapy and/or radiotherapy.

Follow-up	Nil recurrence (14 months)	Nil recurrence (19 months)	Complete clinical response	Nil recurrence (72 months)	Poor prognosis. Referred to palliative care.
